# Integrated analysis of DNA methylome and transcriptome reveals epigenetic regulation of CAM photosynthesis in pineapple

**DOI:** 10.1186/s12870-020-02814-5

**Published:** 2021-01-06

**Authors:** Yan Shi, Xingtan Zhang, Xiaojun Chang, Maokai Yan, Heming Zhao, Yuan Qin, Haifeng Wang

**Affiliations:** 1 State Key Laboratory of Ecological Pest Control for Fujian and Taiwan Crops, College of Plant Protection, Fujian Agriculture and Forestry University, Fuzhou, 350002 Fujian China; 2 Fujian Provincial Key Laboratory of Haixia Applied Plant Systems Biology, Center for Genomics and Biotechnology, Fujian Agriculture and Forestry University, Fuzhou, 350002 Fujian China; 3 College of Horticulture, Fujian Agriculture and Forestry University, Fuzhou, 350002 Fujian China; 4 State Key Laboratory for Conservation and Utilization of Subtropical Agro-Bioresources, Guangxi Key Lab of Sugarcane Biology, College of Agriculture, Guangxi University, Nanning, 530004 Guangxi China

**Keywords:** Epigenetics, DNA methylation, Transcriptome, *Ananas comosus*, Photosynthesis

## Abstract

**Background:**

Crassulacean acid metabolism (CAM) photosynthesis is an important carbon fixation pathway especially in arid environments because it leads to higher water-use efficiency compared to C3 and C4 plants. However, the role of DNA methylation in regulation CAM photosynthesis is not fully understood.

**Results:**

Here, we performed temporal DNA methylome and transcriptome analysis of non-photosynthetic (white base) and photosynthetic (green tip) tissues of pineapple leaf. The DNA methylation patterns and levels in these two tissues were generally similar for the CG and CHG cytosine sequence contexts. However, CHH methylation was reduced in white base leaf tissue compared with green tip tissue across diel time course in both gene and transposon regions. We identified thousands of local differentially methylated regions (DMRs) between green tip and white base at different diel periods. We also showed that thousands of genes that overlapped with DMRs were differentially expressed between white base and green tip leaf tissue across diel time course, including several important CAM pathway-related genes, such as beta-CA, PEPC, PPCK, and MDH.

**Conclusions:**

Together, these detailed DNA methylome and transcriptome maps provide insight into DNA methylation changes and enhance our understanding of the relationships between DNA methylation and CAM photosynthesis.

**Supplementary Information:**

The online version contains supplementary material available at 10.1186/s12870-020-02814-5.

## Background

Drought is one of the most important abiotic stresses affecting the growth and development of plants and crops worldwide [1, 2], resulting in massive production losses. Compared to C3 and C4 plants, Crassulacean acid metabolism (CAM) plants have greater water-use efficiency (WUE) and are better adapted to arid and semi-arid regions.

Pineapple (*Ananas comosus*) is a major tropical crop, representing more than 20% of the world production of tropical fruits [3]. Pineapple fruit is used as a fresh and processed product, and global pineapple production is about 25.8 million metric tons fresh fruit [4]. Pineapple is also a model plant for studying CAM photosynthesis as an adaptation for increased water-use efficiency. In CAM plants, the stomata in the leaves are closed during the day, reducing evapotranspiration, but open at night to absorb carbon dioxide (CO_2_). The CO_2_ absorbed is stored in vacuoles in the form of four-carbon acid malate at night. During the daytime, malate can be transported into chloroplasts and converted into CO_2_ for photosynthesis. The CO_2_ is accumulated near the enzyme Rubisco, which reduces its oxygenase activity [5]. The benefits of this system are that CAM plants reduce water loss by closing stomata during the day and also have increased photosynthetic efficiency. The mechanism and evolution of CAM have been extensively investigated [6–10]. For instance, many genes putatively involved in the carbon fixation module of CAM in pineapple have been identified [6], and many of them are differentially regulated in different leaf tissues [11].

DNA methylation is a heritable epigenetic modification found in most eukaryotic organisms including plants, animals, and fungi [12, 13]. In contrast to animals, where DNA methylation occurs mostly at the CG cytosine sequence context, DNA methylation in plants can occur at CG and CHG (where H is A, T, or C) as well as the CHH context [14–16]. To date, DNA methylome studies in plants have investigated the roles of methylation in seed development, flowering time, hybrid vigor, and gene evolution [17–20]. Many plants are also known to undergo genome-wide DNA methylation changes under different stress and environmental stimuli [2, 21–23]. For example, Liang and colleagues found that genome-wide DNA methylation levels in *Populus* were increased under drought stress compared to the control condition [24]. A recent study in rice highlighted the differences in DNA methylation and the impact on gene expression in three different cultivars (IR64, stress-sensitive; Nagina 22, drought-tolerant; and Pokali, salinity-tolerant). Specifically, extensive differences in DNA methylation among these three cultivars were observed, and numerous differentially methylated regions were found to be associated with differentially expressed genes [25].

In pineapple, DNA methylation was found to play an important role in ethylene-induced flowering [26]. The recently completed genome of pineapple has and will continue to facilitate many areas of research in this species, and the present work focuses on the regulatory impact of DNA methylation on CAM pathway-related genes using temporal and spatial methylome and transcriptome analysis. A comparison of DNA methylation patterns and levels between green and white leaf diel time course allowed us to broadly investigate the epigenetic variation at CAM pathway-related genes. Combined with RNA-seq data, many CAM pathway related genes showed differences in temporal (diel time course) and spatial (green and white leaf) expression, and associated with differentially methylated regions, especially in the CHH context. Taken together, these results suggest a critical role of DNA methylation in the regulation of CAM pathway related genes, and also provide insights into the potential of epigenetic modifications for CAM engineering for the scientific and agronomic community.

## Results

### DNA methylation pathway genes are conserved in the pineapple genome

Much of the knowledge about DNA methylation in plants comes from studies in the model plant *Arabidopsis thaliana*. With the recent availability of the pineapple genome, homolog searches of DNA methylation pathway genes in pineapple are now possible. To identify pineapple genes homologous to *A. thaliana* DNA methylation pathway genes, we searched the annotated protein-coding genes of pineapple by using the BLAST and HMM algorithms. Based on protein similarity and domain conservation, we found that most of the DNA methylation pathway genes in *A. thaliana* are conserved in pineapple, including MET1, CMT2, CMT3, and DRM2, (Table 1). Genes involved in the RNA-directed DNA methylation (RdDM) pathway were also found in pineapple, including AGO4, DCL3, and NRPE5. RNA-seq data were analyzed as an indicator of the functionality of these genes in pineapple, and most of the DNA methylation pathway genes analyzed were expressed at a relatively high level (mean FPKM =10.6; median FPKM=7.1; 10 am green tip sample) in the RNA-seq data. The results therefore indicate that the DNA methylation pathway related genes are retained and functional in pineapple.

**Table 1:** Putative DNA methylation pathway genes in Pineapple

			Pineapple (*Ananas comosus*) expression level (FPKM)									
		Name	Length (a.a)^a^	Locus	4 am		10 am		4 pm		10 pm	
		(Arabidopsis)			Green	White	Green	White	Green	White	Green	White
MET1		VIM1,2,3,4,5,6	645	Aco016212.1	7.46	5.59	3.27	8.46	4.72	7.90	7.54	6.88
		MET1,2a,2b,3	1534	Aco005236.1	1.31	6.52	1.96	4.58	2.42	3.87	1.93	5.87
CMT3		SUVH4	624	Aco004654.1	9.77	19.22	7.51	18.30	9.94	17.35	10.49	18.84
		CMT2	1295	Aco015994.1	2.87	15.82	3.24	18.74	7.69	18.30	4.58	18.97
		CMT3	839	Aco013381.1	0.19	19.10	0.00	11.51	0.29	22.40	0.09	21.01
RdDM	Pol IV recruit	CLSY1/CLSY2	1256	Aco011099.1	0.98	4.92	1.84	4.19	4.69	6.64	3.16	6.52
		SHH1/SHH2	258	Aco017233.1	27.03	21.65	30.40	31.64	23.48	22.87	31.70	22.40
	Pol IV	NRPD1	1453	Aco015559.1	1.16	3.66	4.37	8.40	10.37	7.46	3.21	6.56
	Pol IV+V	NRPD2/NRPE2	1172	Aco009438.1	14.13	36.95	17.12	42.20	22.25	41.79	19.55	44.53
	Pol IV+V	NRPD4/NRPE4	205	Aco018391.1	40.66	93.33	53.21	59.94	55.36	87.71	64.26	78.54
	Pol V	NRPE1	1976	Aco023234.1	9.14	13.25	10.37	14.25	14.42	13.37	16.44	13.77
	Pol V	NRPE5	222	Aco000628.1	9.73	10.40	6.58	17.27	10.27	13.87	9.90	13.93
	Pol V	NRPE9B	114	Aco027798.1	0.00	0.22	0.00	0.00	0.00	0.00	0.00	0.00
	Pol V recruit	DRD1	888	Aco017413.1	6.13	13.41	6.31	14.46	5.84	13.52	7.04	14.76
		DMS3	420	Aco010843.1	14.16	34.50	32.62	47.87	25.58	41.69	22.55	42.65
		RDM1	163	Aco004257.1	10.29	4.50	7.48	2.37	9.67	3.65	7.14	3.46
		SUVH2/9	650	Aco026941.1	5.11	11.78	8.82	15.09	7.62	12.09	7.19	12.68
		RDR2	1133	Aco015280.1	3.58	5.88	2.37	6.57	6.99	7.47	6.29	7.62
		DCL1	1910	Aco016650.1	8.67	20.76	15.73	31.37	20.01	18.71	13.42	22.14
		DCL2	1388	Aco016157.1	10.64	10.61	5.64	38.63	3.61	24.86	6.48	22.66
		DCL3	1580	Aco009891.1	4.10	18.07	8.23	18.51	13.62	17.98	8.24	18.58
		DCL4	1702	Aco008189.1	7.31	5.42	5.36	9.13	7.24	7.44	7.01	7.26
		HEN1	942	Aco008418.1	1.21	2.49	2.54	3.03	2.46	2.48	2.32	3.50
		AGO4	924	Aco017860.1	46.97	108.59	28.67	76.68	60.74	107.18	48.73	115.22
		KTF1	1493	Aco005272.1	20.73	32.16	15.42	33.56	19.91	34.61	23.67	39.20
		IDN2	647	Aco002916.1	10.84	25.56	7.05	19.13	11.89	21.67	11.84	26.22
		SUVR2	740	Aco006945.1	4.65	17.09	5.42	17.02	13.64	20.20	9.10	20.00
		DMS4	346	Aco012515.1	5.56	10.02	6.94	9.22	11.65	11.35	9.82	11.16
		UBP26	1067	Aco013188.1	5.62	8.95	10.84	9.96	10.93	9.95	9.63	9.20
		DRM2	626	Aco007653.1	7.66	12.17	7.38	13.61	4.34	10.75	6.97	12.09
		LDL1	844	Aco002795.1	9.81	5.76	7.29	11.43	10.36	6.00	12.07	6.54
		LDL2	746	Aco009449.1	5.12	6.00	4.22	6.48	3.19	5.43	4.89	5.99
		JMJ14	954	Aco010573.1	9.71	36.65	18.16	28.14	14.35	29.02	13.24	38.39
Others		HDA6	471	Aco016535.1	34.31	15.17	12.77	26.36	13.70	20.04	24.47	21.08
		RDR6	1196	Aco016551.1	2.61	2.88	1.65	6.96	2.61	4.84	4.30	4.50
		MOM1	2001	Aco008982.1	14.63	19.69	18.57	29.89	12.73	20.91	12.70	22.35
		MORC6	663	Aco020316.1	0.03	0.13	0.00	0.00	0.08	0.21	0.29	0.10
		DDM1	764	Aco027111.1	49.64	41.94	41.52	34.79	44.96	39.71	58.30	40.03
		DME	1987	Aco018501.1	9.56	40.31	7.75	31.70	15.71	38.49	10.70	40.59
		ROS1	1393	Aco018808.1	0.17	2.21	0.06	0.57	0.34	2.25	0.33	2.34
		DML2	1332	Aco002923.1	2.49	8.54	5.19	11.25	3.70	9.58	2.84	9.71

^a^ indicates length of the longest protein of this gene family

### Single-base resolution map of DNA methylation in the pineapple genome

**Fig. 1:**
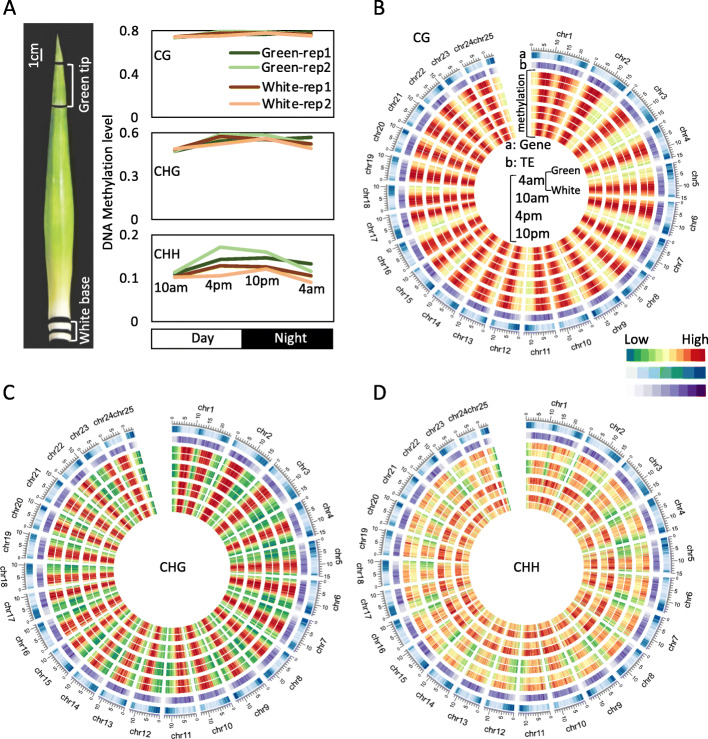
Sampling diagram and DNA methylation profile of pineapple leaf tissue. **a** Pineapple leaf tissue used to survey DNA methylation of CAM photosynthesis-related genes, and genome-wide average DNA methylation comparison between green and white leaf tissue across diel time course. Circos plot of gene density, TE density, and methylation levels of CG (**b**), CHG (**c**), and CHH (**d**) at different time periods between green tip and white base leaf tissue across 25 chromosomes

To explore diel DNA methylation patterns between the photosynthetic (green tip) and non-photosynthetic (white base) leaf tissues of field-grown pineapple, we collected samples at 4 am (ante meridiem), 10 am, 4 pm (post meridiem) and 10 pm across a 24-h period from green tip (photosynthetic) and white base (non-photosynthetic) of leaf tissue and performed whole-genome bisulfite sequencing (WGBS) (Fig. 1). After trimming adapter sequences and filtering low-quality reads, a total of ~ 930 million paired-end reads were generated from the green and white tissue samples across four time course (each with two biological replicates), resulting in ~ 730-fold coverage of the reference genome (Supplemental Table 1). Approximately 70% of all cytosines in the reference genome were covered by at least four reads of different replicates (10 am as an example, Supplemental Figure 1). We also observed an average bisulfite conversion rate of unmethylated C to T of more than 98.7% from the chloroplast control, and the BS-seq data of the two biological replicates of each sample were highly correlated with each other (correlation coefficients> 0.95) (Supplemental Table 2). In summary, our data were reproducible and sufficient for further analysis.

We next investigated the genomic CG, CHG, and CHH methylation levels and found that the genome-wide average DNA methylation levels were very similar between green tip and white base at different times, except for CHH methylation (Fig. 1a). This finding is consistent with data from studies in rice and *Arabidopsis*, in which average DNA methylation of different developmental stages of the same plant is very similar, except for highly specialized tissues, such as the endosperm and the pollen vegetative nucleus [17, 27–29]. The distribution of methyl-cytosine (mC) along the chromosomes was calculated using 500-kb sliding windows with step 100 kb (Fig. 1b-d). Consistent with the findings in other plants, the global DNA methylation profiles for the CG, CHG, and CHH sequence contexts revealed heavily methylated pericentromeric regions (Fig. 1b-d). Unlike the CG and CHG sequence contexts, the CHH methylation of green tip at different times is significant higher than that in white base, especially in pericentromeric regions, where TEs were also enriched (Fig. 1d). We found that the mC distribution in pineapple was different than the previously reported distribution in rice and sorghum [30, 31], and there were also differences between the green and white pineapple leaf tissues (10 am as an example, Supplemental Figure 2). Specially, while pineapple green leaf tissue showed bimodal patterns for all sequence contexts, bimodal patterns were observed only for CG and CHG in pineapple white leaf tissue and in rice and sorghum. This difference suggests that non-CG methylation, specifically, methylation in the CHH context, was maintained more effectively in green tip leaf tissue than in white leaf tissue.

### Photosynthetic and non-photosynthetic pineapple leaf tissues have different DNA methylation patterns in gene and TE regions

**Fig. 2:**
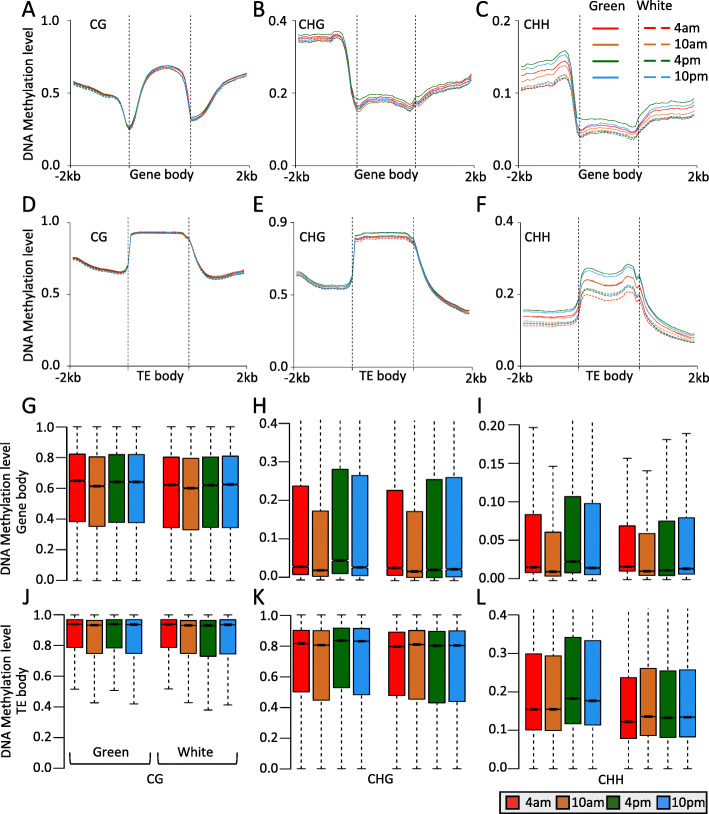
Comparison of DNA methylation patterns of genes and transposable element regions between pineapple green tip and white base leaf. **a**-**c** Metaplot of CG (**a**), CHG (**b**), and CHH (**c**) methylation of gene between green tip and white base across diel time course. **d**-**f** Metaplot of CG (**d**), CHG (**e**), and CHH (**f**) methylation of transposable elements between green tip and white base across diel time course. **g**-**i** Boxplot of CG (**g**), CHG (**h**), and CHH (**i**) methylation of gene between green tip and white base across diel time course. **j**-**l** Boxplot of CG (**j**), CHG (**k**), and CHH (**l**) methylation of transposable element regions between green tip and white base across diel time course

DNA methylation is similar between green tip and white base at different time course in CG and CHG sequence contexts, but very different in CHH context (Fig. 1). To explore the methylation patterns of different genomic structures, we examined the DNA methylation profiles of both gene and TE regions. Consistent to the analysis of genome-wide average DNA methylation, we found that CG DNA methylation is similar of green tip and white base at any time. This phenomenon is consistent in the gene and TE regions (Fig. 2a and d). Significantly, non-CG DNA methylation is very different of green tip and white base across different time, especially CHH DNA methylation (Fig. 2b-c and e-f). The CHH DNA methylation of green tip is significantly higher than that of white base, and its shows temporal rhythm changes (methylation is increased at 4 pm and 10 pm, but decreased at 4 am and 10 am), suggesting that it may be related to the photosynthesis of pineapple leaves, such as the CAM pathway.

To further investigate diel DNA methylation levels of gene and TE regions, we focused on gene and TE body regions rather than flanking regions. From the temporal DNA methylation data, we compared the DNA methylation levels of gene and TE regions in green and white leaf tissue at different time, and observed diurnal methylation changes of both gene and TE regions. All contexts DNA methylation decreased in the early morning (10 am) and increased at the afternoon (4 pm) of both green and white leaf tissues across different time (Fig. 2g-j), except CHG and CHH DNA methylation of TE regions in white leaf tissue (Fig. 2k-l). Similar results were found in upstream and downstream regions of gene (Supplemental Figure 3). Consistent to the metaplot analysis of gene and TE regions, we found non-CG DNA methylation of green tip is higher than that of white base, especially CHH context. Collectively, our results showed that diel DNA methylation patterns and levels of both green tip and white base, and CHH methylation should play a critical role into this day-night cycling.

### Association between DNA methylation and gene expression

**Fig. 3:**
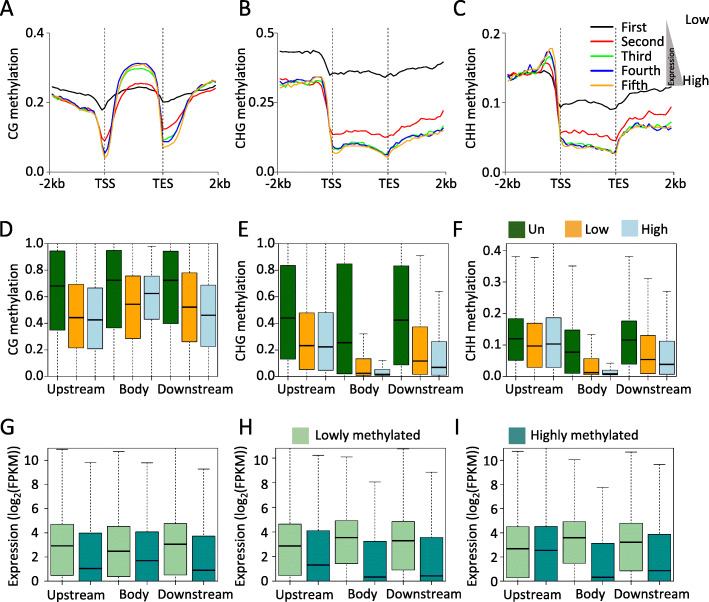
Correlation analysis between gene expression and DNA methylation. **a**-**c** Comparative analysis between gene expression and DNA methylation for the CG (**a**), CHG (**b**), and CHH (**c**) sequence contexts. **d**-**f** DNA methylation analysis of differential expression genes for the CG (**d**), CHG (**e**), and CHH (**f**) sequence contexts. **g**-**i** Expression analysis of different methylated genes in the contexts of CG (**g**), CHG (**h**), and CHH (**i**). Upstream, gene body and downstream regions were shown

It is well established that DNA methylation is associated with gene expression [16, 32]. Moderately expressed genes have more methylation than lowly or highly expressed genes, and promoter DNA methylation is usually negatively correlated with gene expression, except in some cases in which it promotes gene expression [18, 33–35]. To investigate DNA methylation regulation of gene expression in pineapple leaf tissues, we initially examined the CG, CHG, and CHH methylation levels of all expressed genes in green leaf tissues (take 10 am as an example). Expressed genes were proportionally divided into five groups according to the level of gene expression: genes with the lowest expression composed the first group, and the most highly expressed genes composed the fifth group. Consistent with previous studies in other plant species, moderately expressed genes were the most highly methylated in gene body regions for the CG sequence context, i.e., the fourth group of genes was the most highly methylated in our study (Fig. 3a) [16, 18]. Non-CG methylation was highest in the first group of genes in both the gene body and flanking regions, and the second groups of genes also had higher non-CG methylation than the remaining groups of genes. These data suggest that non-CG methylation has a role in repressing gene expression (Fig. 3b-c).

We also observed that CG, CHG, and CHH methylation levels in promoter and downstream regions were highest in the most lowly expressed genes. To further examine the relationship between DNA methylation and gene expression, we analyzed the DNA methylation levels of three groups of genes (unexpressed, lowly expressed, and highly expressed genes) at different genic regions (promoter, gene body, and downstream regions). Compared to unexpressed genes, lowly and highly expressed genes had significantly lower CG, CHG, and CHH methylation at all three genic regions (Mann-Whitney test, *P*-value< 0.001) (Fig. 3d-f). Additionally, the methylation levels of lowly expressed genes were significantly higher than those of highly expressed genes at the different genic regions, except for CG methylation in the gene body and CHH methylation in the promoter region. A recent study in cassava also positively correlated gene body CG methylation with gene expression [18]. Thus, based on the present analysis and studies in other plant species, gene body CG methylation may be positively associated with gene expression [36]. We next focused on lowly and highly methylated genes, and examined the expression level differences between them. Similar to the results described above, lowly methylated genes were more highly expressed than highly methylated genes, and this was consistent for all three sequence contexts in the three genic regions except for promoter CHH methylation (Mann-Whitney test, *P*-value < 0.001) (Fig. 3g-i). Together, these data suggest that the relationship between DNA methylation and gene expression depended on the genic regions, as well as the cytosine sequence context.

### Diel DNA methylation regulation of pineapple leaf

**Fig. 4:**
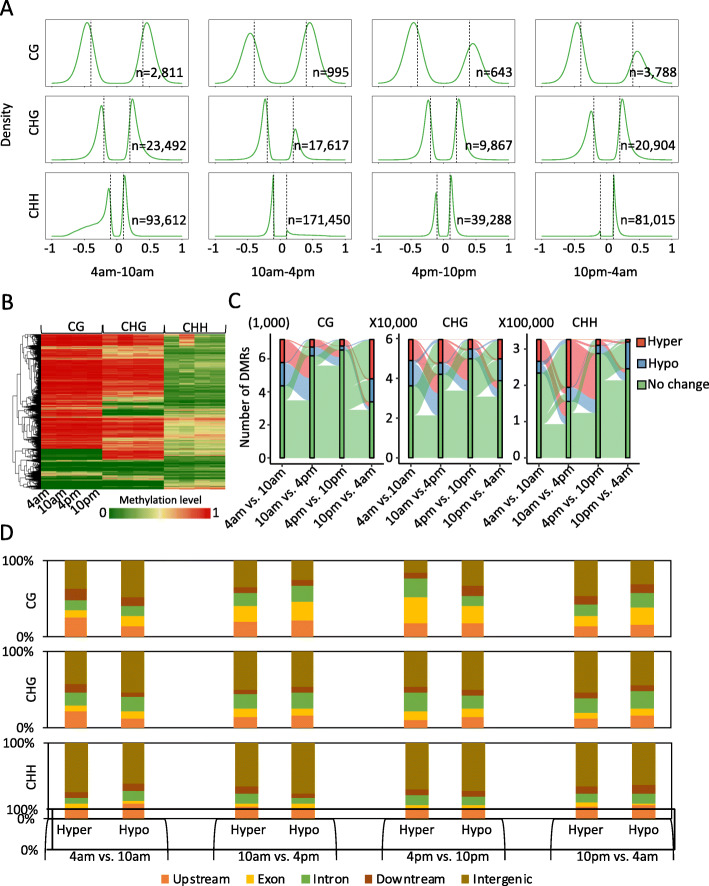
DNA methylation variance of green tip at different diel periods. **a** Density of differential methylation regions of CG, CHG, and CHH contexts across diel time course of green tip leaf tissue. ‘n’ denotes the number of DMRs. The vertical dotted lines mean the defined threshold of different DNA methylation changes (e.g. 0.4 for CG, 0.2 for CHG, and 0.1 for CHH methylation). **b** Heat map of DNA methylation levels of all DMRs (CG+CHG+CHH) of green tip leaf tissue. **c** Sankey plot of DMR dynamics across different periods of green tip of CG, CHG, and CHH contexts. **d** The genomic distribution of DMRs of green tip across different diel time course

Genome-wide DNA methylation levels and patterns in green tip leaf tissue were similar to those in white base leaf tissue. To investigate diel DNA methylation patterns of photosynthetic leaf tissue, we firstly calculated average weighted DNA methylation levels for each sequence context using 100-bp windows [37] and identified regions with significantly different methylation levels between different diel time course of green leaf tissues (hereafter referred to as differentially methylated regions, DMRs). For continuous time comparison, we found that the number of differentially methylated regions of CHH type is the largest, followed by CHG type and CG type. This also implies that the role of CHH methylation in the regulation of circadian rhythm of green leaf tissue is more important than CG and CHG methylation (Fig. 4a). In addition, we found that CHH DNA methylation changes were greatest of 10 am to 4 pm during the day, and showed hypermethylation at 4 pm compared with 10 am. To further examine the association between DNA methylation changes across different diel time course in three sequence contexts (CG, CHG and CHH), we compared DMRs of different sequence contexts, and found that DMRs are not overlapped of three sequence contexts. For example, the hypermethylated regions of CHH at 10 am may not necessarily be hypermethylated on CG and CHG sequence contexts (Fig. 4b); these CHH hypermethylated regions of 10 am become hypomethylated regions or no methylation changes regions at other times. This phenomenon is also consistent in CG and CHG methylation (Fig. 4c). This finding is consistent with the DMRs comparison between green tip and white base or white base at different diel periods (Supplemental Figure 4). Our analysis indicates that DNA methylation changes are dynamic of green tip across different periods, and probably play an important role in this diel cycle, especially CHH context.

We next examined the genomic characteristics of regions that changes of DNA methylation across diel time course. We tested the extent of overlap between DMRs within gene promoters (2 kb upstream of transcriptional start site), exons, introns, downstream regions of genes and intergenic regions. Although differential DNA methylation occurs in many locations, we found that most DMRs are enriched in intergenic regions. In addition, the DMR-enriched regions differ greatly between different comparisons of various time courses (Fig. 4d). For example, Aside from DMRs in intergenic regions, DMRs were most enriched in promoter regions of comparison between 4 am to 10 am, while exons were most enriched between 4 pm and 10 pm. This result is consistent with the above comparative analysis of DMRs, which shows the dynamic changes of DNA methylation in the green tip during the diel time course.

### Characterization of DMR-associated differential expression genes

**Fig. 5:**
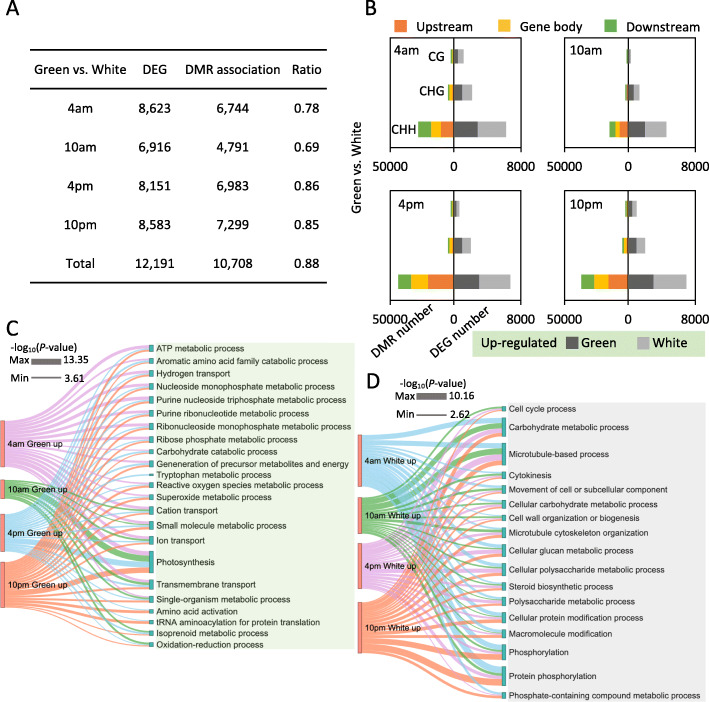
Differential methylation regions associated DEGs analysis. **a** Summary of DEGs and DMR-associated DEGs between green tip and white base at different diel periods. **b** The number of DMR-associated DEGs. DMRs were divided into up−/down-stream and gene body regions of CG, CHG and CHH contexts. DEGs were divided in to up- and down-regulated DEGs. **c** Enriched GO terms of DMR-associated up-regulated DEGs in green tip of different time course. **d** Enriched GO terms of DMR-associated up-regulated DEGs in white base of different time course. *P*-value was scaled to the thickness of line

To investigate the impact of DNA methylation variance on gene expression differences of pineapple leaf tissues, we generated RNA-seq libraries for the same tissues used in the DNA methylation analysis and identified differentially expressed genes (DEGs) (Supplemental Table 3). We also compared our transcriptome data with the transcriptome data from previous studies, and found that our data is highly reproducible and consistent with each other (Supplemental Figure 5, 6]. We first focused on differentially expressed genes across different diel time courses between green tip and white base. Approximately 12,191 DEGs were identified across different time stages, and the most differentially expressed genes were identified at 4 am (8623) and the least were at 10 am (6916) (Fig. 5a). For these DEGs, we found that a large number of DEGs are associated with differential methylation regions (DMRs). For example, there are 10,708 (87.8%, 10,708 out of 12,191) DEGs overlapped with DMRs, most of which are CHH-type DMRs (Fig. 5b). This result is consistent with green tip and white base (Supplemental Figure 6 and Supplemental Figure 7). The results demonstrate that a large number of genes are differentially expressed across diel time course and that diel DNA methylation changes are critical for this transcriptional dynamics of pineapple leaf.

We defined DMR-associated genes as those overlapping with a DMR in the 2000 bp upstream or 2000 bp downstream region or within the gene body. 5778, 2710, and 10,708 DMR-associated DEGs at different times were identified in green tip, white base and comparison between green tip and white base, respectively. To understand the critical roles of DMR-associated DEGs for pineapple leaf tissue of diel time periods. We performed Gene Ontology (GO) term enrichment analysis of DMR-associated DEGs from different periods comparison between green tip and white base. For DMR-associated DEGs in comparison between green tip and white base, we divided these genes into up-regulated genes in green tip or up-regulated genes in white base, and performed GO categories analysis separately. The results showed that the two groups of genes had very different functional categories. The DMR associated up-regulated DEGs in green tip were significantly enriched in the pathway of photosynthesis, transmembrane, and ion transport (Fig. 5c). On the contrary, DMR associated up-regulated DEGs in white base were enriched in protein phosphorylation, carbohydrate metabolic and microtubule-based process (Fig. 5d).

For DMR-associated DEGs in green tip across different periods, genes involved in transmembrane transport, photosynthesis, light harvesting, ion homeostasis processes were highly enriched (Supplemental Figure 6). For DMR-associated DEGs in white base, GO functional analysis showed an enrichment of genes associated with photosynthesis, oxidation-reduction process, and carbohydrate metabolic process (Supplemental Figure 7). Our data suggest that DNA methylation changes regulate gene expression difference between green and white leaf tissue and probably involved in regulating the source-sink relationship of the CAM photosynthesis process.

### CAM pathway related genes regulated by DNA methylation

**Fig. 6:**
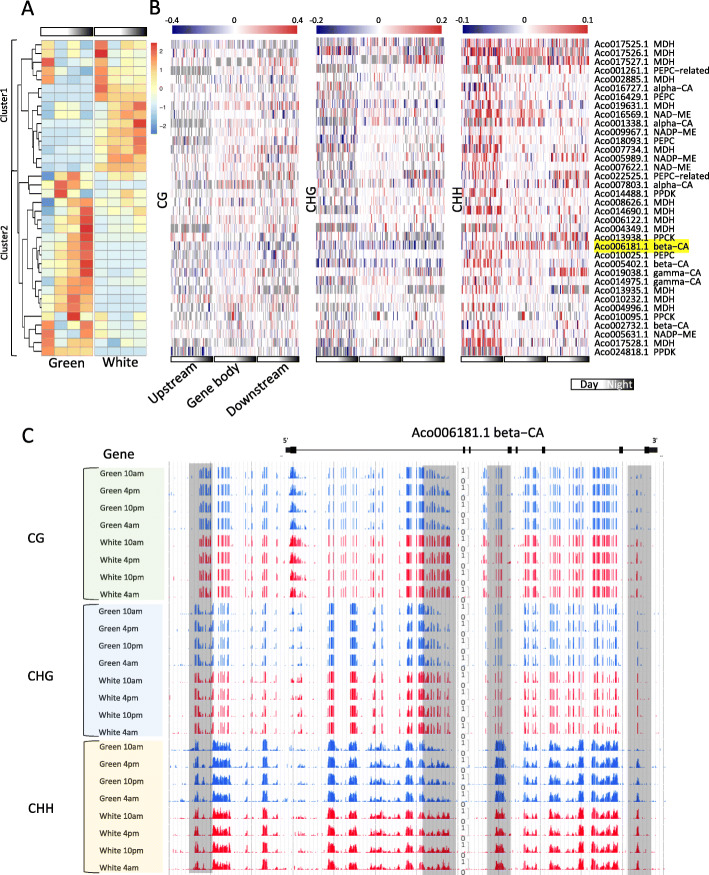
Diel expression patterns and DNA methylation of CAM pathway-related genes. **a** Expression patterns of CAM related genes for the diel time course between green tip and white base. Two clusters were grouped by hierarchical clustering. **b** DNA methylation dynamics across CAM related genes between green tip and white base at different time course (From 10 am to 4 pm, and then 10 pm to 4 am). Upstream, gene body and downstream regions were divided into 20 bins separately, and then calculated the methylation level of each bin. **c** Genome browser snapshot showing DNA methylation changes of Aco006181.1 (beta-CA) of green tip across different diel time course of CG, CHG, and CHH contexts

Because the GO analysis of DMR-associated DEGs indicated an enrichment of genes with major regulatory roles in photosynthesis, light signaling, carbohydrate metabolic process and transmembrane transport pathways (Fig. 5, Supplemental Figure 6 and Supplemental Figure 7), we next focused on genes that are known to play critical roles in the CAM pathway. Previous studies have identified that 38 genes are involved in the carbon fixation module of pineapple CAM process through homologous search and expression profile analysis [6]. We found that the majority of them are differentially expressed in green and white leaf tissues at different diel periods, and divided into two clusters. Genes of cluster 2 showed rhythmic expression in green tip but low expression in white base, and genes of cluster 1 showed that diel expression patterns in white base but arrhythmic expression in green tip (Fig. 6a). Most of these CAM genes are DMR-associated DEGs between green and white leaf tissues at different diel periods, including carbonic anhydrase (CA), phosphoenolpyruvate carboxylase (PEPC), malate dehydrogenase (MDH), phosphoenolpyruvate carboxylase kinase (PPCK) and pyruvate orthophosphate diakineses (PPDK) (Fig. 6b, Supplemental Table 4), majority of them also have been verified by our qRT-PCR validation (Supplemental Figure 8 and Supplemental Table 5). CA genes are responsible for carbon dioxide fixation in CAM photosynthesis. Three beta-CA and two gamma-CA genes are highly expressed in photosynthetic green tip, but lowly expressed in non-photosynthetic white base. Previous studies have shown that only beta-CA genes could be major protein for carbon fixation rather than other CA genes. In present study, we identified a greater number of CHH type DMRs located across gene body and flanking regions of all three beta-CA genes. For example, we identified several DMRs located in the Aco006181.1 (beta-CA) gene body region, and these regions showed reduced DNA methylation in green tip at different diel periods compared to white base (Fig. 6c). We suspected that these DMRs should be related to the gene expression difference of beta-CAs between green tip and white base.

In addition, we also found that many genes involved in CAM-related pathway are regulated by DNA methylation, such as transporters, glycolysis and gluconeogenesis. Several genes were studied to be candidates for pineapple to hydrolyze vacuolar sucrose to hexose, and showed peak expression at early or late morning, such as Aco023030.1, Aco023036.1 and Aco017533.1. All these three members of vacuolar acid invertase gene family showed diel expression patterns and associated many DMRs between green tip and white base (Supplemental Table 7). Aco005379.1, which is a candidate for a vacuolar hexose exporter, and was previously shown highly expressed in green tissue, but did not show significant day-night cycling [11]. But we found this gene was highly expressed in green tip with peak expression at night (10 pm). A total of 56 DMRs located across gene body and flanking regions of Aco005379.1, and most of them are CHH type DMRs (Supplemental Figure 9, Supplemental Table 6).

In pineapple genome, there are nine enzymes involved in glycolysis and gluconeogenesis. All of these nine enzymes shown higher expression in green tip than that in white base [11], and most of them are expressed rhythmically in green tip and arrhythmic expression in white base. But the regulatory mechanisms are still unclear. We found that all these nine enzymes were overlapped with many DMRs. For example, there are 30 DMRs across Aco024971.1 (triose-phosphate isomerase) gene body and flanking regions (Supplemental Table 7). Many studies have shown that circadian clock-related genes and some transcription factors encoding genes play essential roles in the photosynthesis pathway. We also analyzed the expression changes of pineapple circadian related genes and MADS-box transcription factor genes between green and white tissues during the diel time, and found that many of their expression were regulated by the changes of DNA methylation (Supplemental Table 8 and 9) [38, 39]. Taken together, these results suggest that a large number of genes involved into CAM-related pathway are regulated by DNA methylation.

## Discussion

In this study, we generated temporal and spatial genome-wide single-base resolution DNA methylome maps and transcriptome profiles of pineapple leaf tissues, which greatly enhance and complement previous knowledge of CAM pathway studies. Comparing the methylation levels in photosynthetic green tip and non-photosynthetic white base leaf tissues revealed no significant global differences in CG and CHG methylation, but CHH methylation was significantly reduced in white base leaf tissue compared with green tip leaf tissue across different diel time course. In addition to the large number of DMRs located in the intergenic regions, we found that many DMRs were overlapped with gene body and flanking regions. Previous studies have shown that promoter methylation is often associated with downstream gene repression, but the role of gene body DNA methylation is still controversy. However, recent study have shown that gene body DNA methylation can alter gene expression [40]. There are obvious dynamic local DNA methylation changes during diel time course of green tip or white base of pineapple leaf. We hypothesized that dynamic DNA methylation changes in green tip or white base leaf tissue should be related to the CAM-related pathway. Through sampling diel DNA methylation patterns in both green and white pineapple at different diel time course, we were able to identified differential methylation changes related to the CAM photosynthetic pathway. By combining DNA methylation data and transcriptome data, we could identified a large number of DMR-associated DEGs, which are often enriched in several important biological pathways of CAM cycle, such as photosynthesis, lighting harvest, carbohydrate metabolism, transporter and protein phosphorylation. There are three CA gene families annotated in pineapple genome (alpha-CA, beta-CA, and gamma-CA), but only beta-CAs were expressed highly in green tip, and showed diel expression patterns [6]. We found all three beta-CAs in pineapple genome showed different expression between green tip and white base tissues, and were associated with many DMRs, especially CHH contexts.

Many of the presently available research on CAM pathways has focused on evolutionary and transcriptome analyses of CAM pathway-related genes [7, 11]. In *Guzmania monostachia,* people found that the up-regulated genes of the leaf tip are mainly enriched in the regulation of stomatal movement, tryptophan metabolic process, chlorophyll biosynthetic process, and aspartate metabolic process. However, the up-regulated genes of leaf base are mainly related to response to water deprivation, starch, and sucrose metabolic processes [41]. These results are consistent with our findings, indicating that core CAM-related genes and steps between inducible and constitutive CAM plants are similar.

Bromeliad leaves are described as showing a morpho-physiological gradient from the apex to the base [42–44]. In addition, previous studies of leaf segment RNA-seq data in pineapple, rice, and maize all suggest that fructose transporter (SWEET17) plays an essential role in exporting fructose from leaf sheath. They proposed that leaf tip and base within the same pineapple leaf play the role of sink and source cycle [11]. In our transcriptome data, we found that many genes involved in sucrose transporter, hexose transporter, glycolysis and gluconeogenesis were also associated with different methylation divergence, such as Aco005379.1, Aco023036.1, Aco005368.1 and Aco024987.1. We believed that DNA methylation plays a critical role of sink and source cycles daily between leaf tip and base by regulating the expression level of these transporters encoding genes. Gene duplication and expansion was initially proposed to be the driven force for the evolution of the CAM pathway [45]. However, it is still controversial, many studies have shown that CAM pathway should be evolved by differential expression of CAM-related genes or neofunctionalization rather than gene dosage [7, 10, 11].

## Conclusions

DNA methylation has been long recognized as a mechanism for gene expression regulation, repetitive element silencing, and plays a critical role for plant development and stress response. Here we showed that spatial and temporal DNA methylation and transcriptome changes during pineapple leaf CAM cycle. Our results strongly suggest that the transcription regulation of many key CAM-regulated genes involves DNA methylation and provide epigenetic insights for engineering of CAM in other crop improvement.

## Methods

### Plant material, library construction and sequencing

In this study, plant material of *Ananas comosus* var. *comosus* cultivar MD-2 was acquired from Guangxi Academy of Agricultural Sciences (GAAS), China (22.84^。^N, 108.48^。^E). Identification of the plant materials was made by the GAAS and original plant was acquired from Del Monte company (www.delmonte.com). The voucher specimen was deposited at the herbarium of the College of Plant Protection, Fujian Agriculture and Forestry University, China. DNA was extracted from two regions (green tip and white base) of ‘D’ leaf of pineapple (*Ananas comosus* var. *comosus* cultivar MD-2) using Qiagen DNeasy Plant Mini Kit, and BS-seq libraries were prepared using the TruSeq Nano DNA LT kit (Illumina), as described previously [32]. For each tissue type, two libraries corresponding to two biological replicates were sequenced on a HiSeq X Ten system (Illumina) to obtain paired-end 150-bp reads per the manufacturer’s instructions.

Total RNA was extracted from the same tissue used for the BS-seq libraries, and RNA-seq libraries were prepared using the TruSeq Preparation Kit with polyA mRNA selection, per the manufacturer’s instructions (Illumina). Three libraries (only two libraries for 10 am sample) were pooled and sequenced to obtain paired-end 150-bp reads on the Illumina HiSeq X Ten system.

### BS-seq data analysis

For each biological replicate of BS-seq data, bisulfite-converted reads were trimmed by Trimmomatic and aligned to the pineapple reference genome using BSMAP v2.90 [46]. Four mismatches were allowed per 100-bp read length, and only uniquely mapped reads were kept for further analysis. The conversion rate was estimated using one minus the average DNA methylation level from chloroplast genome. Correlation between two biological replicates was calculated using the average DNA methylation level of non-overlapping 100-bp windows, and the *cor()* function in R software (www.r-project.org). Metaplots for both gene and TE regions were generated using the average weighted DNA methylation level of three separate regions: the upstream region (100-bp bin size), gene body (20 proportional bins), and downstream region (100-bp bin size). Differentially methylated regions (DMRs) were determined by DMRcaller, which is a versatile R/Bioconductor [47].

### RNA-seq data analysis

RNA-seq reads were trimmed by Trimmomatic and aligned to the pineapple reference genome by HISAT2 v2.1.0 [48] with default parameters, and only uniquely mapped reads were kept. Expression value was quantified by StringTie v1.3.3b [48] as FPKM (fragments per kilobase per million mapped reads). Differentially expressed genes (DEGs) were identified by DESeq2 v1.22.2 with default parameters [49]. RNA-seq data of Ray M. et al. (2015) were downloaded from NCBI BioProject PRJNA305042, and only data at 4 am, 10 am, 4 pm and 10 pm time points were considered for comparisons. The same RNA-seq analysis pipeline in present work was performed on these public dataset, genes with FPKM > 0.5 were considered as expressed, and used for heatmap and Pearson correlation coefficient analysis.

### qRT-PCR validation

Total RNA for real-time PCR analysis was extracted following manufacturer’s protocol using RNA extraction kit (Omega Bio-Tek, Shanghai, China) from “D” leaf that was the same stage for BS-seq and RNA-seq analysis. Green tissue at the leaf tip and white tissue at the leaf base of the same leaf were collected and quickly froze in liquid nitrogen at 10:00 am, 4 pm, 10 pm, and 4 am as Fig. 1a shown. These sample were stored in − 80 °C refrigerator until total RNA extraction. Total RNA was diluted with nuclease-free water. 1 μg of purified total RNA was reverse transcribed to cDNA in a 25 μl reaction volume using M-MLV reverse transcriptase (Promega) according to the supplier’s instructions. The qRT-PCR reactions were carried out with Quantitative kit (TRANS, Beijing, China) with the program: 95 °C for 30s; 40 cycles of 95 °C for 5 s and 60 °C for 30s; 95 °C for 15 s. For each condition, three technical replicates and at least three independent biological replicates were performed. The PCR primers were listed in Supplemental Table 5.

### GO enrichment analysis

GO enrichment of DMR-associated genes was performed by using the OmicShare tool, free online platform for data analysis (www.omicshare.com/tools) with hypergeometric test. Only GO terms with *P*-value less than 0.01 were used for further analysis.

## Supplementary Information

**Additional file 1.**

## Data Availability

The data reported in the present paper were deposited into the Gene Expression Omnibus (GEO) database (accession number GSE120401).

## Abbreviations

CAM: Crassulacean acid metabolism
RdDM: RNA-directed DNA methylation
GO: Gene ontology
WGBS: Whole genome bisulfite sequencing
FPKM: Fragments per kilobase per million mapped reads
DMRs: DNA methylation regions
DEGs: Differential expression genes
