# Supplementary material for: Integrated analysis of DNA methylome and transcriptome reveals epigenetic regulation of CAM photosynthesis in pineapple
Source: BMC Plant Biol. 2021 Jan 6;21:19. doi: 10.1186/s12870-020-02814-5 (PMC7789485; doi:10.1186/s12870-020-02814-5)
Supplement: Supplementary file 1 — Additional file 1. [file 12870_2020_2814_MOESM1_ESM.zip › Supplemental Table 1-3,5 Supplemental Fig.1-9.pdf]

Supplemental Table 1. Summary of BS-seq reads mapping.

		Alignment reads	Total reads	Coverage (X)	Conversion rate	Mapping ratio	mCG	mCHG	mCHH
4am_rep1	Green	98,974,967	137,410,204	28	98.98%	72.03%	79.07%	56.79%	13.35%
4am_rep2		83,187,376	120,146,056	27	98.68%	69.24%	76.46%	52.03%	11.42%
4am_rep1	White	99,217,842	138,941,906	30	98.86%	71.41%	75.87%	52.11%	10.71%
4am_rep2		84,556,478	119,798,650	29	98.89%	70.58%	74.91%	48.94%	9.04%
10am_rep1	Green	82,981,674	122,114,678	27	99.49%	67.95%	73.56%	47.19%	11.04%
10am_rep2		81,001,302	117,737,248	29	99.40%	68.80%	73.32%	47.29%	11.36%
10am_rep1	White	84,563,465	124,177,904	27	99.48%	68.10%	74.28%	48.41%	10.28%
10am_rep2		89,809,353	128,548,624	34	99.41%	69.86%	74.22%	48.80%	10.39%
4pm_rep1	Green	70,655,331	99,713,640	39	99.22%	70.86%	76.68%	54.43%	14.48%
4pm_rep2		67,594,264	99,454,308	33	99.26%	67.97%	78.66%	58.71%	17.16%
4pm_rep1	White	76,261,200	111,042,076	39	99.49%	68.68%	77.63%	57.40%	12.86%
4pm_rep2		72,896,596	105,981,410	33	99.48%	68.78%	75.37%	51.74%	10.66%
10pm_rep1	Green	67,882,311	99,780,136	33	99.21%	68.03%	77.15%	55.34%	14.69%
10pm_rep2		75,093,428	109,428,896	32	99.26%	68.62%	78.95%	58.08%	16.06%
10pm_rep1	White	68,603,873	99,641,912	33	99.18%	68.85%	77.37%	55.50%	12.50%
10pm_rep2		87,403,767	129,479,154	35	99.20%	67.50%	77.83%	55.96%	12.12%

Supplemental Table 2. Pearson correlation coefficient between the two biological replicates of BS-seq sample.

Pearson correlation coefficient	CG	CHG	CHH
Green(4am)	0.97	0.98	0.93
White(4am)	0.97	0.98	0.94
Green(10am)	0.99	0.99	0.97
White(10am)	0.99	0.99	0.97
Green(4pm)	0.96	0.98	0.95
White(4pm)	0.97	0.98	0.95
Green(10pm)	0.98	0.99	0.97
White(10pm)	0.98	0.99	0.97

Supplemental Table 3. Summary of RNA-seq reads mapping.

	Sample		Mapping reads	Total reads	Mapping ratio
Green	4pm	rep1	43,318,861	47,752,244	90.72%
	4pm	rep2	56,397,824	62,056,284	90.88%
	4pm	rep3	59,619,354	65,698,144	90.75%
White	4pm	rep1	49,309,178	55,857,152	88.28%
	4pm	rep2	60,106,442	69,157,454	86.91%
	4pm	rep3	42,230,085	49,111,332	85.99%
Green	10pm	rep1	44,903,956	49,385,338	90.93%
	10pm	rep2	45,391,014	49,770,040	91.20%
	10pm	rep3	54,122,072	60,097,110	90.06%
White	10pm	rep1	45,374,449	52,826,964	85.89%
	10pm	rep2	46,012,034	52,880,314	87.01%
	10pm	rep3	48,409,686	57,702,546	83.90%
Green	4am	rep1	70,719,566	77,242,172	91.56%
	4am	rep2	50,968,946	56,914,326	89.55%
	4am	rep3	49,848,312	55,800,816	89.33%
White	4am	rep1	44,249,356	51,281,830	86.29%
	4am	rep2	57,824,558	65,961,376	87.66%
	4am	rep3	50,664,431	57,356,832	88.33%
Green	10am	rep1	38,256,368	46,223,158	82.76%
	10am	rep2	37,324,314	45,092,100	82.77%
White	10am	rep1	35,484,112	44,021,882	80.61%
	10am	rep2	44,221,272	54,522,034	81.11%

Supplemental Table 5. The qRT-PCR primers.

Gene	Common name	Forward primer	Reverse primer
Aco007803.1	Alpha-1	CCACATGGTACACAAGAGTGAT	CTCTTCGCGCCTCCTTATTT
Aco016727.1	Alpha-2	CTGCTCGCATCATCCTTTCT	GCCGGTGGATCCATCATTATATC
Aco001338.1	Alpha-3	CATTACCACCATCTCCCTCATC	GCTCCCTTGAACGTAGCTAAA
Aco002732.1	Beta-1	TGGCTTGAGCCAAGAAATCA	CCAGAAACCTGTACTCCAACTC
Aco006181.1	Beta-2	GCGATAGAGTATGCGGTCATC	GATGGACATGAGCCCTTTGA
Aco005402.1	Beta-3	CTGTGCGAAACATAGCAAACA	CAAGGTGGAGGACAGCATATT
Aco014975.1	gamma-1	CGTAGGGACGAGGAGTATGA	TGAAGTATGTTGTCGGGAAGG
Aco023760.1	gamma-2	CTGCTGGAGCCCTTGTATG	GTTGCGGACTGGGTGATAAA
Aco017803.1	gamma-3	CACGAGGAGACTCTGGAGAT	TAAGCAGTGGAGTAGGGAAGA
Aco010025.1	PEPC-1	AGTTTGAGTCCTGGCGATTC	CTGCTTGTTCCTCCTCCTAAAT
Aco018093.1	PEPC-2	GCAACACCGGAGATGGAATA	CATGGGATTGCACGTAGAGAT
Aco016429.1	PEPC-3	CGATGAACTGAGGCGTCATAA	CACGACGTAGATAGTGTGGAATAG
Aco022525.1	PEPC-4	GCTCTTTACGAGAGCTTCAGAG	GGTTCCTTCTGCTGTGGATAC
Aco001261.1	PEPC-5	GCGATGTCTCCTCCAGAATAAC	AATCTAGTGTTAGGTACTGCCTTTAG
Aco010095.1	PPCK-1	ATGAGCGAGGGCTTGAAC	CGAGAAGCAGCGGAAGAC
Aco013938.1	PPCK-2	TTGAGGAGGGAGTACGAGAT	CCTTCTCCACCGACTTCAC
Aco024818.1	PPDK-3	AGGAGTGGGAGAGCTCATTA	CACCATGCTCACCACAAATTC
Aco014488.1	PPDK-4	TCGGAGAGAGGGTTGTCTAAA	GCCTGCTTTACGATCTCCATTA
Aco006122.1	MDH-1	CTTATGTCCAGTCGACCATCAC	CAATCCAAGCACCTCCTCTAC
Aco007734.1	MDH-2	CTGCGATCATCAAAGCAAGAAA	GTGTCCCAAGAACCCAATCA
Aco013935.1	MDH-3	GTCTCTGATGATGACTGGCTAAA	CAGCACTTGCAGCAGATAAAG
Aco002885.1	MDH-4	TCTGATTGCGAATGGTGTATCA	CACTTTCGGGTGCTTCTTCT
Aco004349.1	MDH-5	GGTGGGCATGCTGGTATAA	CCTCTTAGTGAGCGCCTTAAT
Aco014690.1	MDH-6	GAGGTTGTGGAAGCGAAAGA	ATCTCCGTCGAGTGCTCTAA
Aco017525.1	MDH-7	CCTTCTTCCGCCAGATCAAA	GAGATCTTGATGCGGTGGTT
Aco017526.1	MDH-8	TTAGTGCCGGATCCGATGA	CGTCGACGTGCTCACCTA
Aco017527.1	MDH-9	CAGCTTCTCCGGCTTCG	TGAACGGAGAGTAGGAGGTT
Aco017528.1	MDH-10	TGAAGAAGACCTCCTCTCTCTC	TCTTTGATCTGGCGGAAGAAG
Aco019631.1	MDH-11	TGCAGCAGAAGCCCTAAAT	AGTGCAAGCTTCAACCAAATC
Aco010232.1	MDH-12	CGGACCGGATCAACCAATAA	CCTCCCTCAACAAAGGGTAAA
Aco004996.1	MDH-13	GTCACGGAGCTTCCATTCTT	CCAGCCCTCTCAAACTCATT
Aco008626.1	MDH-14	GGTCAATCCTCCGTGTTCTT	GGCACTTAAATCCTTGCATGAG
Aco009967.1	NADP-1	TAAGGGCCGTGCAATCTTT	CCGAAACCCGGGAATATGTAG
Aco005631.1	NADP-2	CCAGAAGAGCATCCAGGTTATT	CAGCGCCTCATTGTTTGTTC
Aco005989.1	NADP-3	GTTGCCAGTGCTTACCAATTAC	CTCTTCTTTGCCTCAACCCTAT
Aco016569.1	NADP-4	CCTATAGTTTACACGCCCACTG	CCCACGATCTTCTGCACTAAA
Aco007622.1	NADP-5	GTTGCACTAGCTGGACTACTT	GCTCCTACTACGACAATCTTCTG

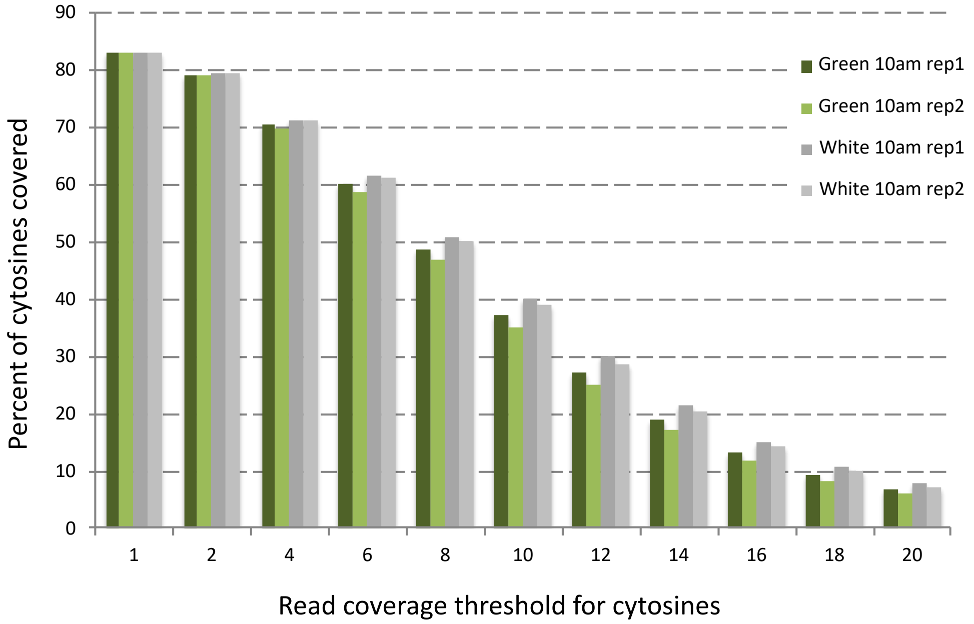

Supplemental Figure 1. Genome-wide cytosine coverage by mapped BS-seq data. As figure shown, about 70% of total cytosines were covered by at least 4 mapped reads in each replicates of green and white leaf tissue at 10 am.

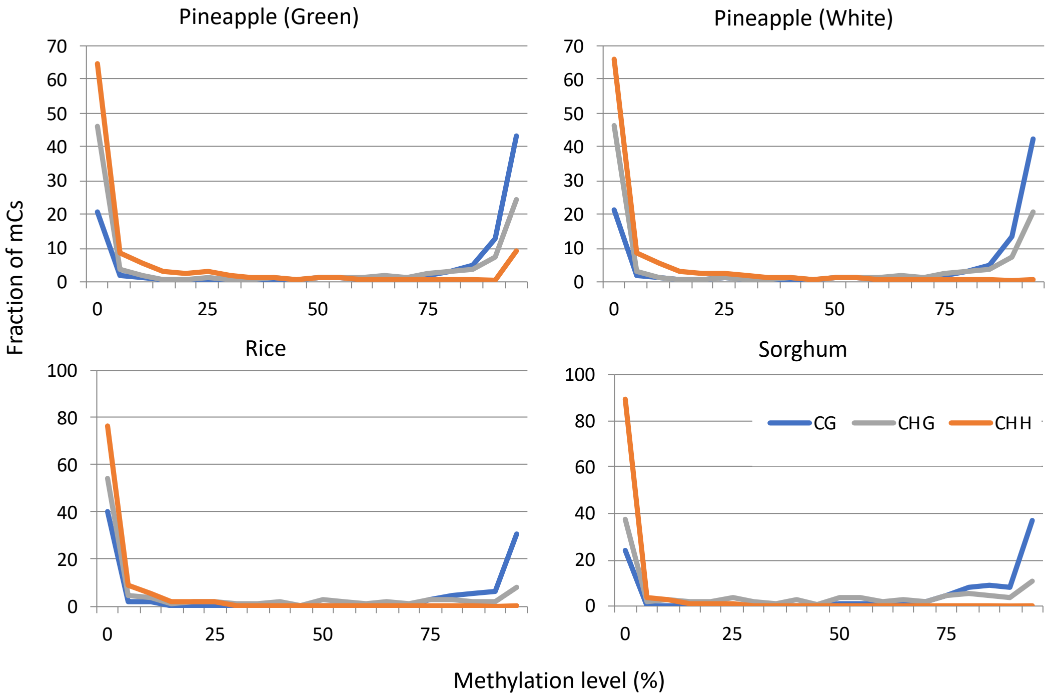

Supplemental Figure 2. The DNA methylation level distribution between pineapple (green tip and white base), rice and sorghum. Only methyl-cytosines covered by at least 3 reads were counted and used for methylation calculating.

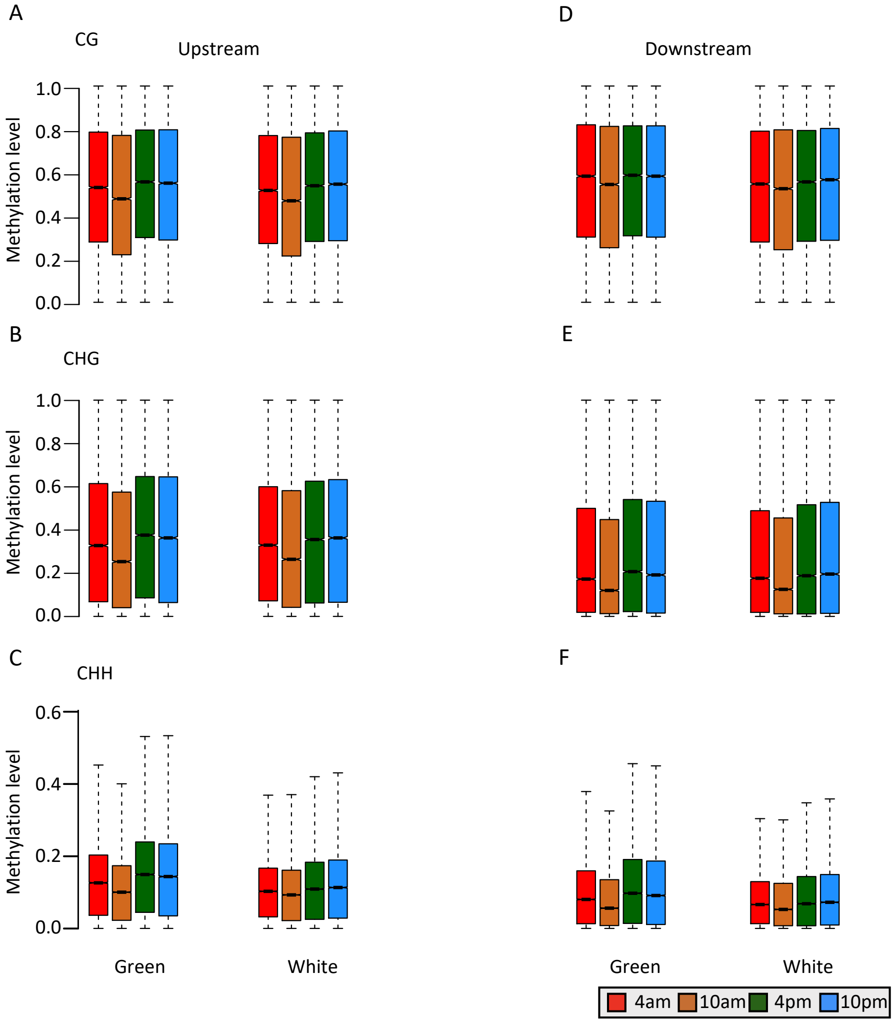

Supplemental Figure 3. DNA methylation patterns of genes regions for the diel time course in green tip and white base. (A-C) DNA methylation patterns across gene upstream regions of green tip and white base across different diel time course. (D-F) DNA methylation patterns across gene downstream regions of green tip and white base across different diel time course.

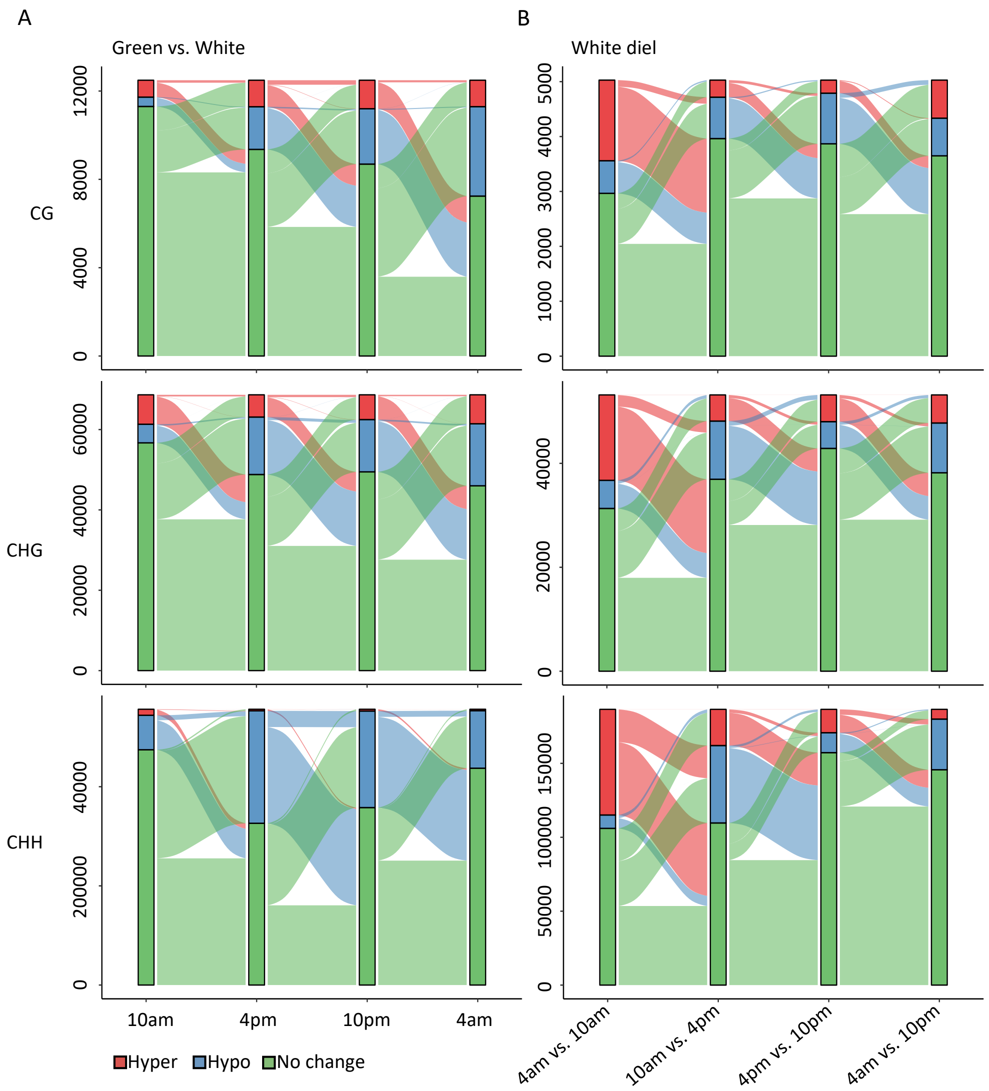

Supplemental Figure 4. DMRs dynamics of pineapple green tip and white base leaf tissues at different periods. (A) Sankey plot of DMR dynamics across different periods between green tip and white base. (B) Sankey plot of DMR dynamics across different periods of white base. DMRs of CG, CHG, and CHH contexts were shown.

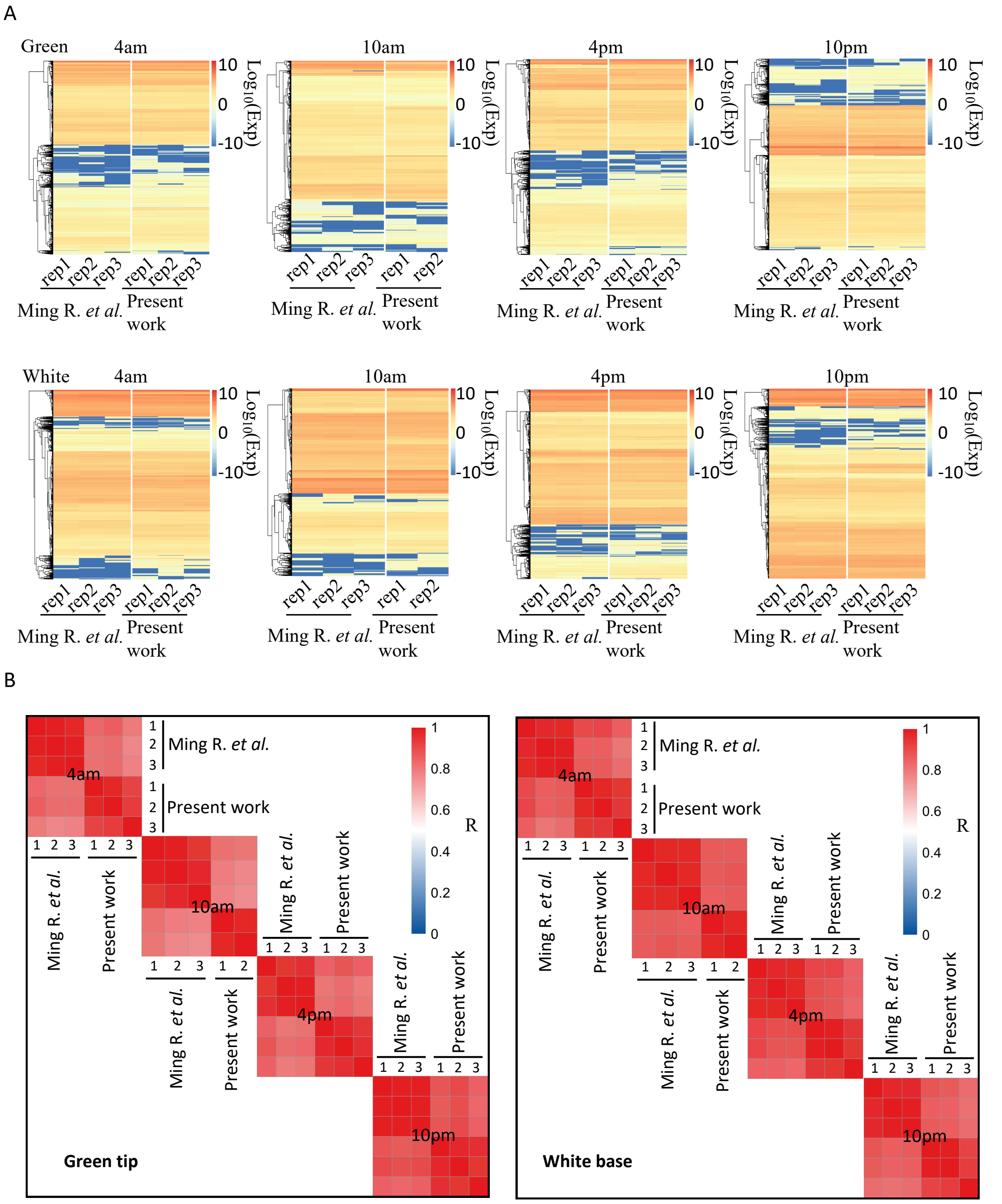

Supplemental Figure 5. Correlation comparison between our transcriptome data and Ray M. et al (2015). (A) Heat map showing hierarchical clustering of expression value between different replicates of data in present work and Ray M. et al. (2015). (B) Pearson correlation coefficient among our data and Ray M et al. (2015) data at different time periods of green and white leaf tissues.

A

Green diel DEG and DMR	DEG number	DMR associated DEG	ratio
4amvs10am	4,470	2,915	0.65
10amvs4pm	3,831	2,971	0.78
4pmvs10pm	1,570	696	0.44
10pmvs4am	2,850	1,970	0.69
Total	7,541	5,778	0.77

B C

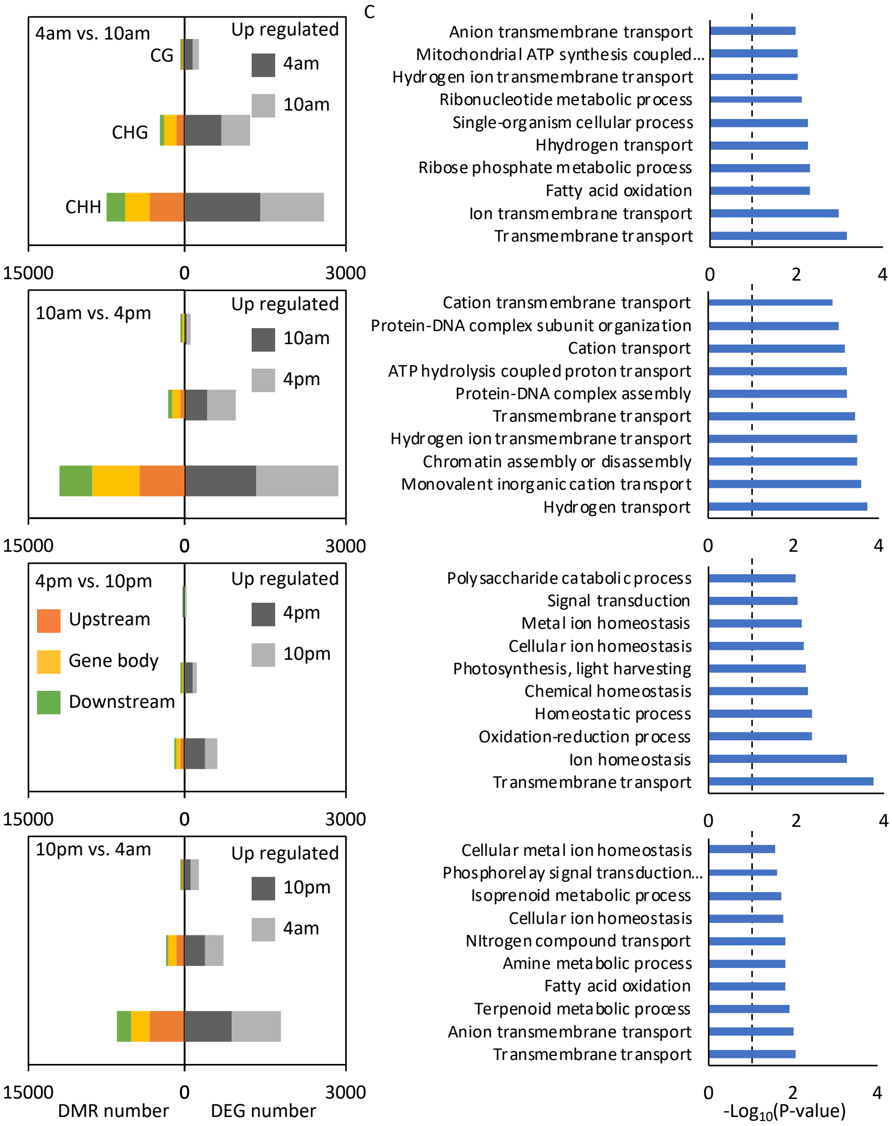

Supplemental Figure 6. Differential methylation regions associated DEGs analysis of green tip. (A) Summary of DEGs and DMR-associated DEGs of green tip at different diel periods. (B) The number of DMR-associated DEGs. DMRs were divided into up-/down-stream and gene body regions of CG, CHG and CHH contexts. (C) Enriched GO terms of DMR-associated DEGs of different time course.

A

White diel DEG and DMR	DEG number	DMR associated DEG	ratio
4amvs10am	3,056	1,916	0.63
10amvs4pm	2,743	1,662	0.61
4pmvs10pm	101	30	0.30
10pmvs4am	318	159	0.50
Total	3,941	2,710	0.69

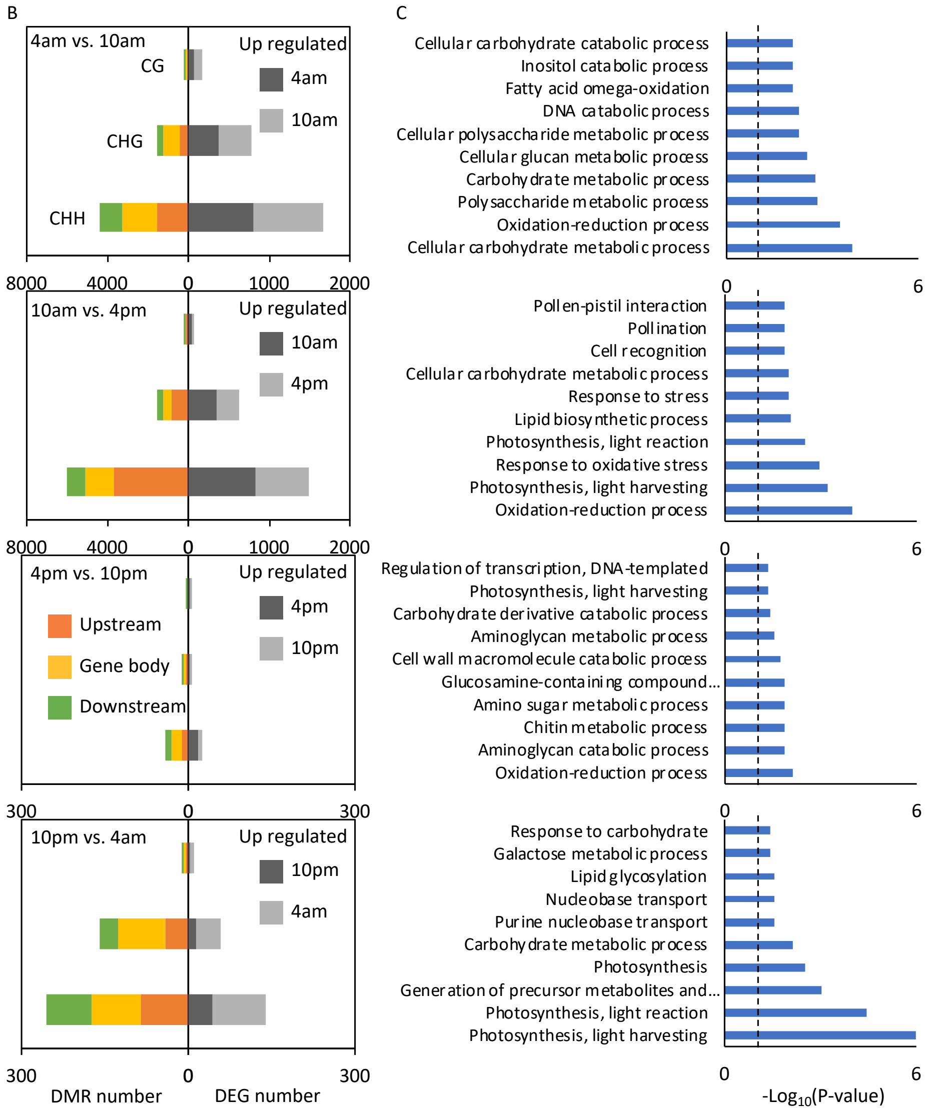

Supplemental Figure 7. Differential methylation regions associated DEGs analysis of white base. (A) Summary of DEGs and DMR-associated DEGs of white base at different diel periods. (B) The number of DMR-associated DEGs. DMRs were divided into up-/down-stream and gene body regions of CG, CHG and CHH contexts. (C) Enriched GO terms of DMR-associated DEGs of different time course.

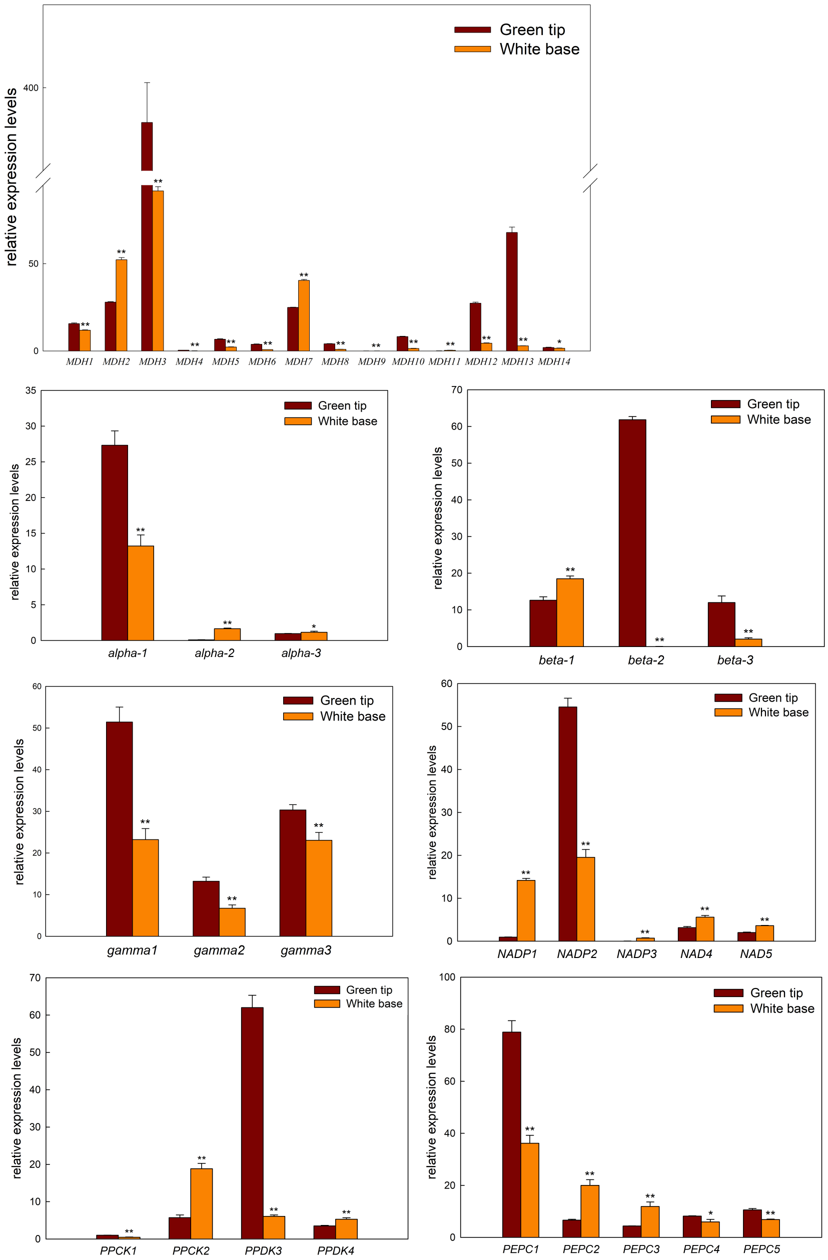

Supplemental Figure 8. qRT-PCR validation of 37 selected CAM pathway related genes. Double stars indicate P value <0.01, and single star indicates P value <0.05 (Student's t-test).

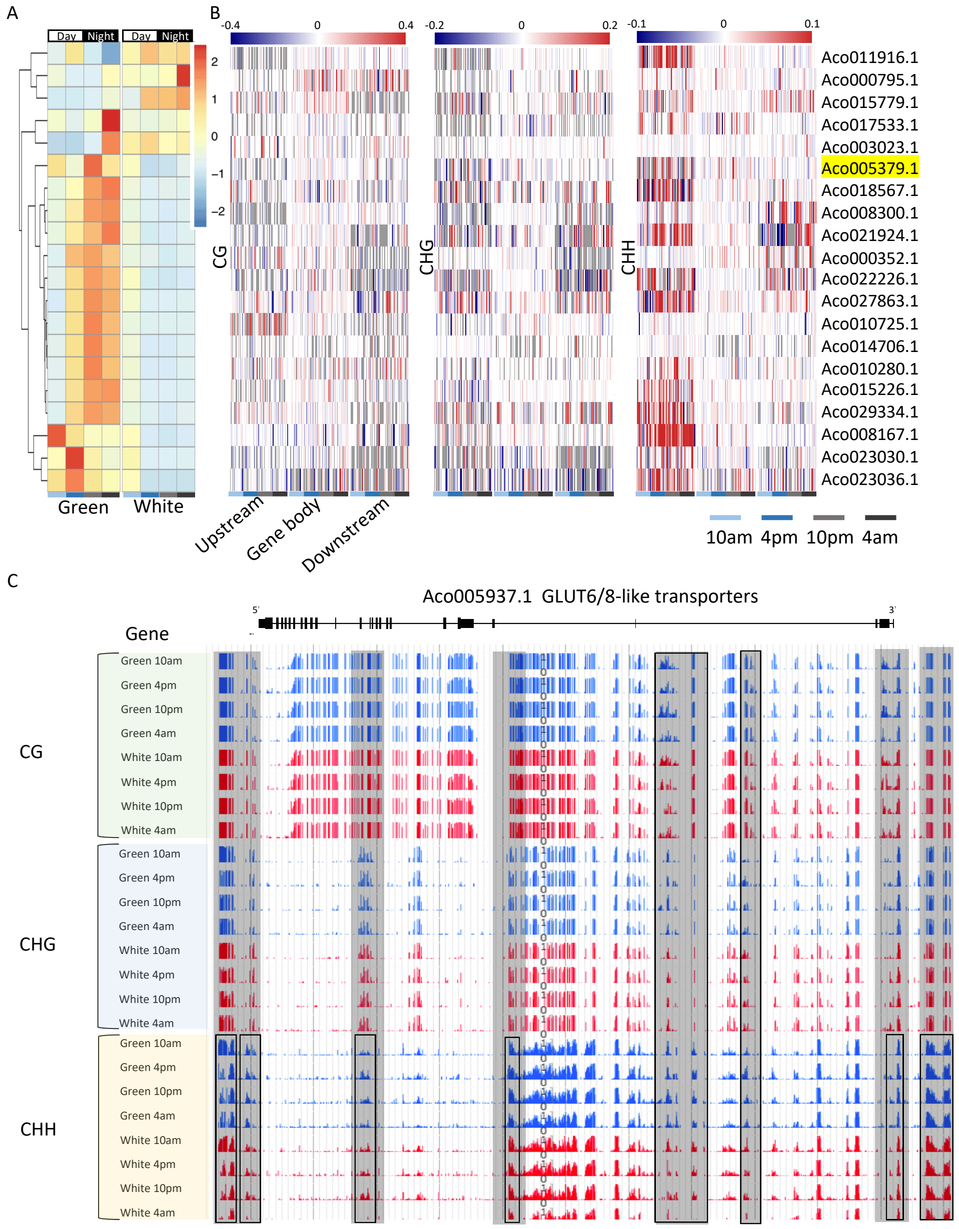

Supplemental Figure 9. Diel expression patterns and DNA methylation of CAM related transporter genes. (A) Expression patterns of CAM related transporter genes for the diel time course in green tip and white base. (B) DNA methylation divergence across CAM related transporter genes between green tip and white base at different time course. Upstream, gene body and downstream regions were divided into 20bins, and methylation levels of each bin were calculated. Methylation divergence between green tip and white base was calculated by Green-White. (C) Genome browser snapshot of DNA methylation changes of Aco005937.1 (GLUT6/8-like transporter).
